# Exploring racial and ethnic disparities in medication adherence among Medicare comprehensive medication review recipients

**DOI:** 10.1016/j.rcsop.2021.100041

**Published:** 2021-06-25

**Authors:** Xiaobei Dong, Chi Chun Steve Tsang, Jim Y. Wan, Ya-Chen Tina Shih, Marie A. Chisholm-Burns, Samuel Dagogo-Jack, William C. Cushman, Lisa E. Hines, Junling Wang

**Affiliations:** a Department of Clinical Pharmacy and Translational Science, University of Tennessee Health Science Center College of Pharmacy, 881 Madison Avenue, Room 214, Memphis, TN 38163, United States of America; b Department of Clinical Pharmacy and Translational Science, University of Tennessee Health Science Center College of Pharmacy, 881 Madison Avenue, Room 212, Memphis, TN 38163, United States of America; c Department of Preventive Medicine, University of Tennessee Health Science Center College of Medicine, 66 N. Pauline, Suite 633, Memphis, TN 38163, United States of America; d Department of Health Services Research, University of Texas MD Anderson Cancer Center, 1515 Holcombe Blvd., Unit 1444, Houston, TX 77030, United States of America; e Department of Clinical Pharmacy and Translational Science, University of Tennessee Health Science Center College of Pharmacy, 881 Madison Avenue, Room 264, Memphis, TN 38163, United States of America; f Division of Endocrinology, Diabetes & Metabolism, Clinical Research Center, University of Tennessee Health Science Center College of Medicine, 920 Madison Avenue, Suite 300A, Memphis, TN 38163, United States of America; g Department of Preventive Medicine, University of Tennessee Health Science Center College of Medicine, 66 North Pauline Street, Suite 651, Memphis, TN 38163, United States of America; h Pharmacy Quality Alliance, 5911 Kingstowne Village Parkway, Suite 130, Alexandria, VA 22315, United States of America; i Department of Clinical Pharmacy & Translational Science, University of Tennessee Health Science Center College of Pharmacy, 881 Madison Avenue, Room 221, Memphis, TN 38163, United States of America

**Keywords:** Medicare Part D medication therapy management, Comprehensive medication review, Racial and ethnic disparities, Disparity decomposition, Medication adherence, Multiple chronic conditions, AHRF, Area Health Resources Files, Asians, Asians and Pacific Islanders, BO, Blinder-Oaxaca decomposition method, CMR, Comprehensive Medication Review, CMS, Centers for Medicare & Medicaid Services, HPSA, Health Professional Shortage Area, MBSF, Master Beneficiary Summary File, MSA, Metropolitan Statistical Area, MTM, Medication Therapy Management, Part D, Medicare prescription drug benefits, PDC, Proportion of Days Covered, PQA, Pharmacy Quality Alliance, Star Ratings, Parts C and D Star Ratings, VRDC, Virtual Research Data Center, Whites, Non-Hispanic Whites

## Abstract

**Background:**

There has been a lack of evidence on whether there are racial and ethnic disparities in medication nonadherence among individuals receiving comprehensive medication review (CMR), a required component of the Medicare Part D medication therapy management (MTM) services.

**Objectives:**

To explore racial/ethnic disparities in medication nonadherence among older MTM enrollees who received a CMR and to determine how much the identified disparities can be explained by observed characteristics.

**Methods:**

The retrospective study used 100% of the 2017 Medicare claims, including MTM data. Linked Area Health Resources Files provided community characteristics. Nonadherence was defined as proportion of days covered <80%, and was measured for diabetes, hypertension, and hyperlipidemia medications. Racial/ethnic disparities were examined by logistic regressions that included racial/ethnic minority dummy variables. A nonlinear Blinder-Oaxaca decomposition method was applied to decompose the identified disparities.

**Results:**

Compared with non-Hispanic Whites (Whites), Blacks were respectively 39% (odds ratio [OR] = 1.39, 95% confidence interval [CI] = 1.33–1.45), 27% (OR = 1.27, 95% CI = 1.22–1.32), and 43% (OR = 1.43, 95% CI = 1.39–1.47) more likely to be nonadherent to diabetes, hypertension, and hyperlipidemia medications; Hispanics were 20% (OR = 1.20, 95% CI = 1.14–1.27) more likely to be nonadherent to hyperlipidemia medications. The total portion of disparity explained was 13.42%, 7.66%, 14.87%, and 10.69% respectively for disparities in Black-White (B–W) diabetes, B–W hypertension, B–W hyperlipidemia, and Hispanic-White hyperlipidemia. The top three contributors were the proportion of married-couple families, census region, and male gender.

**Conclusions:**

A lower level of community affluence and social support, regional variations, and a lower proportion of males in Blacks and Hispanics may contribute to the disparities in medication nonadherence. The large unexplained portion of the disparity attests that nonadherence is a complex issue. The Medicare MTM program needs to implement measures to reduce disparities in medication adherence.

## Highlights

• This retrospective study used 100% of the 2017 Medicare claims including MTM data.
• Racial/ethnic disparity in medication adherence was identified among CMR recipients.
• Blacks were more likely than Whites to be nonadherent to all medications studied.
• Hispanics were more likely than Whites to be hyperlipidemia medication nonadherent.
• Observed characteristics explained close to 15% of the identified disparity.

## 1 Introduction

Medication nonadherence is a serious issue in the U.S. and has been found to be associated with adverse health outcomes, increased mortality, and excessive health care costs.^1^^,^^2^ Medication nonadherence is one of the common medication therapy problems experienced by adults age 65 and older,^3^ who tend to have multiple chronic conditions that necessitate complex medication regimens.^4^ Among the 59.9 million Americans that Medicare covered in 2018, 85% aged 65 or above,^5^ and two-thirds of them had multiple chronic conditions.^6^ The proportion of older beneficiaries in fee-for-service Medicare programs who were nonadherent to diabetes, hypertension, and hyperlipidemia medications was respectively 35%, 25%, and 38%.^7^ The annual healthcare cost saving for Medicare was estimated at $23 billion if those nonadherent beneficiaries became adherent.^7^

To promote appropriate medication adherence, the Centers for Medicare & Medicaid Services (CMS) has incorporated adherence measures for diabetes, hypertension, and hyperlipidemia medications in its Star Ratings quality evaluation program for Medicare Part D drug plans.^8^ These measures were developed by the Pharmacy Quality Alliance (PQA), a non-governmental nonprofit established initially as a public-private partnership by CMS following the implementation of the Medicare Part D prescription drug benefit in 2006.^9^ Apart from the adherence-specific metrics, CMS adopted medication therapy management (MTM) as a required Part D component that targets beneficiaries having multiple chronic conditions, taking multiple Part D prescription medications, and incurring high medication expenditures.^10^

While conceived as a sweeping strategy to optimize medication use, improve health outcomes, and reduce healthcare costs, the MTM program has been under-utilized with its average enrollment rates remaining as low as around 10%.^11^ CMS has attempted to increase the MTM enrollment by measures such as lowering eligibility thresholds; however, such attempts were only met with limited success.^10^^,^^12^ Studies suggested that minorities may not be able to fully benefit from the program because its eligibility criteria are utilization-based whereas minorities tend to consume fewer medications and have lower drug expenditures.^13^, ^14^, ^15^ A crucial obstacle for a meaningful MTM program reform is the lack of evidence of the program's actual effects, particularly those on minorities. The only evaluation conducted thus far reported improved medication adherence among MTM-enrolled patients with diabetes, chronic obstructive pulmonary disease (COPD) and congestive heart failure (CHF); however, the study did not examine the program's effects on minorities.^16^ The findings from that study were consistent with those from non-Medicare MTM studies, including studies that examined services provided by pharmacists in communities, which generally observed MTM’s positive effect on medication adherence. However, evidence was inconclusive due to a wide latitude in study designs, methodology, and statistical power.^17^^,^^18^ The lack of evidence of the effects of MTM on minorities, whether in Medicare or non-Medicare setting, may be related to the limited availability of data,^17^ since commercial datasets generally do not include racial/ethnic information and Medicare MTM data were not available until recently.^19^

This study aimed at using the newly available Medicare MTM data to explore racial/ethnic disparity in medication adherence among older Medicare beneficiaries with a focus on those having received a comprehensive medication review (CMR), which is a required component of the MTM program and operationalized as an annual consultation mostly provided by a pharmacist to the beneficiary or the beneficiary's caregiver.^20^ Since 2016, the CMR completion rate, i.e. the percent of Part D beneficiaries enrolled in the MTM program who received a CMR during a measurement year, has been a performance measure of Star Ratings for Part D plans.^8^ Given that CMR has been assigned a key role in medication management, it is essential to examine whether there is any room for improvement for the CMR program. Particularly, it is critical to determine whether racial/ethnic disparities exist among CMR recipients. Previous research reported a positive effect of CMR or targeted medication review, a related but different Medicare MTM service, on adherence by comparing recipients with non-recipients.^16^^,^^21^ A study focusing on only CMR recipients found that adherence was improved in certain types of patients; however, the study sample was small (*N* = 97) and limited to one state.^22^ While recent literature has documented racial/ethnic disparities in adherence and CMR receipt,^3^^,^^23^^,^^24^ it is unknown whether racial/ethnic disparity exists in medication adherence among CMR recipients and, if such disparity does exist, what observed characteristics may have contributed to the disparity. The objectives of this study included the following: (1) to determine whether there was racial/ethnic disparity in medication adherence among older Medicare MTM enrollees who received a CMR. The medications of interest included those for diabetes, hypertension, and hyperlipidemia. The 3 conditions were chosen because they are the targeted conditions in the Star Ratings adherence measures^8^; and (2) subject to findings from the first objective, to determine the extent to which the observed disparity can be explained by characteristics included in our regression model, with the use of an extension of the Blinder-Oaxaca (BO) decomposition method.^25^

## 2 Methods

### 2.1 Data sources and study sample

The study used 100% of the 2017 Medicare data including Parts A and B claims, Part D Drug Event (PDE) File, Part D MTM Data File, and the Master Beneficiary Summary File (MBSF) base segment. Beneficiary information obtained from the data files included those on diagnostic records, prescription medication claims, date of MTM enrollment, date of CMR receipt, and demographic information including age, gender, and race/ethnicity.^26^ To supplement the individual-level factors with neighborhood characteristics, the Medicare data were linked to the Area Health Resources Files (AHRF) using the beneficiary county of residence information in the MBSF. County-level information such as population education, income, insurance, and healthcare capacity was obtained from AHRF.^27^

The study sample was limited to MTM-enrolled beneficiaries who were 65 years of age or older, had continuous coverage for fee-for-service Parts A, B, and D plans, received a CMR, and had at least one of the 3 diseases of interest, namely diabetes, hypertension, and hyperlipidemia. The diseases were identified based upon beneficiaries' prescription claims according to the PQA technical specifications.^28^ In addition, beneficiaries needed to meet PQA inclusion criteria for diabetes, hypertension, and hyperlipidemia adherence measures: having at least 2 fills of the medications for a disease of interest on different days and the first fill occurring at least 91 days before the end of the year. Beneficiaries who were in hospice care or had end-stage renal disease were excluded.^28^ Likewise, those with claims for insulin were excluded from the diabetes measure and those with claims for sacubitril/valsartan were excluded from the hypertension measure.^28^ Beneficiaries were categorized into the following 5 racial/ethnic groups: non-Hispanic Whites (Whites), Blacks, Hispanics, Asians and Pacific Islanders (Asians), and all other races/ethnicities (Other).

### 2.2 Outcome measure

Following PQA's technical specifications, the present study used the proportion of days covered (PDC) to determine medication adherence. Specifically, the PDC was calculated as follows: the denominator was the number of days counting from the date of the first fill after CMR receipt through the last day of 2017. The denominator also represented the treatment period. The numerator was the number of days covered by at least 1 prescription medication in the medications for the disease of interest specified by PQA during the treatment period.^28^ Prescription medications for the diseases of interest were identified based upon the list of medications in the PQA technical specifications for each disease-specific adherence measure.^28^ Medications in the list were matched with those in the PDE File using the National Drug Codes in both the PQA list and the PDE File. A beneficiary's medication fill history was then examined in the latter. A PDC of less than 80% was considered as nonadherence.^28^ For each of the 3 diseases of interest, a binary outcome variable was constructed with the value of 1 representing nonadherence (i.e., PDC < 80%) and the value of 0 indicating otherwise.

### 2.3 Conceptual framework

The Gelberg-Andersen Behavioral Model for Vulnerable Populations was used as the theoretical framework for selection of covariates, which were categorized into predisposing, enabling, and need factors.^29^ The predisposing factors are demographic and contextual characteristics that predict the likelihood of medication utilization. Covariates in this category included age, gender, race/ethnicity, and the following county-level characteristics: proportion of married-couple families, proportion of people with education at or above high school level, income per capita, and proportion of people without health insurance. The enabling factors, which represent means and resources, were operationalized by county-level covariates including metropolitan statistical area (MSA), health professional shortage area (HPSA), and census regions. The need factors encompass self-perceived or objectively evaluated health, measured by a risk adjustment summary score calculated using the hierarchical condition category risk adjustment model that CMS uses in payment adjustment to plans based on beneficiary demographic and diagnostic characteristics.^30^ A higher score indicates a probability of a beneficiary having higher healthcare expenditures, which suggest a worse health status.

### 2.4 Statistical analysis

In descriptive analyses, the characteristics of beneficiaries were compared across racial/ethnic groups. For each group, the number and proportion for each categorical variable and mean and standard deviation for each continuous variable were obtained. The differences across groups were then examined by Chi-square tests for categorical variables and *t*-tests for continuous variables. To explore racial/ethnic differences in outcome measures, the number and proportion of beneficiaries in each racial/ethnic group who were nonadherent to medications were first obtained for each of the conditions of interest. Chi-square tests were then conducted to compare unadjusted differences across groups. Adjusted differences were examined by multivariate logistic regressions that included a dummy variable for each racial/ethnic minority group. The Whites served as the reference group for race/ethnicity dummy variables, whose coefficients were the estimates of interest. If a race dummy variable's estimate, namely an odds ratio (OR), was greater than 1 and statistically significant, it would indicate that the subject minority group had a higher odds of nonadherence compared to Whites and therefore the result would suggest disparity. Because the regression model included county-level covariates, clustered standard errors at the county level were used to account for potential within-county correlation.

### 2.5 Disparity decomposition

Identified disparities were decomposed using a nonlinear extension of the Blinder-Oaxaca (BO) decomposition method. Initially developed to explain racial and gender disparities in wage, the BO method partitions the mean outcome difference between 2 groups into 2 portions: one that is explained by covariates, or observed characteristics, and the other that is unexplained, or due to unobserved characteristics.^31^^,^^32^ The original BO technique is used in linear regressions with continuous outcome variables, but has been extended for applications in nonlinear regressions with categorical outcome measures.^25^ Following Fairlie's (2005) approach, the study decomposed the disparity in the following procedure.^25^

First, the logistic regression model was run to obtain coefficient estimates, which were then used to calculate the predicted probability of having the outcome for each observation in the Whites and subject minority group. Next, a random subsample of Whites was drawn to form a sample of Whites of the same size as the full minority sample. After each observation in the 2 cohorts was separately ranked by their predicted probabilities, they were matched 1 to 1 based on the rankings. The matching was to ensure that the distributions of the characteristics of the White subsample and minority sample were comparable to each other. To estimate each covariate's contribution to the total disparity, the average predicted probability for the White subsample was calculated, followed by a sequential replacement of the White distribution with the minority distribution for each covariate, one at a time, while the distributions of all other covariates were kept constant. After each replacement, the average predicted probability was again obtained for the White subsample. The change in the average predicted probability following the replacement of the White distribution with the minority distribution for a covariate quantified the unique contribution of that covariate.

The estimates obtained from the above procedure were dependent on the characteristics of the randomly drawn White subsample. To address this, the study followed Fairlie (2005) and drew multiple random subsamples of Whites, separately calculated the decomposition estimates, and averaged the estimates. While Fairlie used 1000 random subsamples, this study tested with a smaller number given the massive sample of the entire Medicare population. The mean estimates beyond 30 subsamples were found to be very similar; thus, the decomposition results reported in this paper were based on average values obtained from 30 random subsamples of Whites.

All statistical analyses were conducted using SAS®9.4 (SAS Institute, Inc., Cary, NC) and the statistical significance level was set a priori at 0.05. The Medicare data were accessed via the CMS Virtual Research Data Center. The Institutional Review Board at the corresponding author's institution approved the study (approval number #17–05326–XM).

## 3 Results

**Fig. 1:**
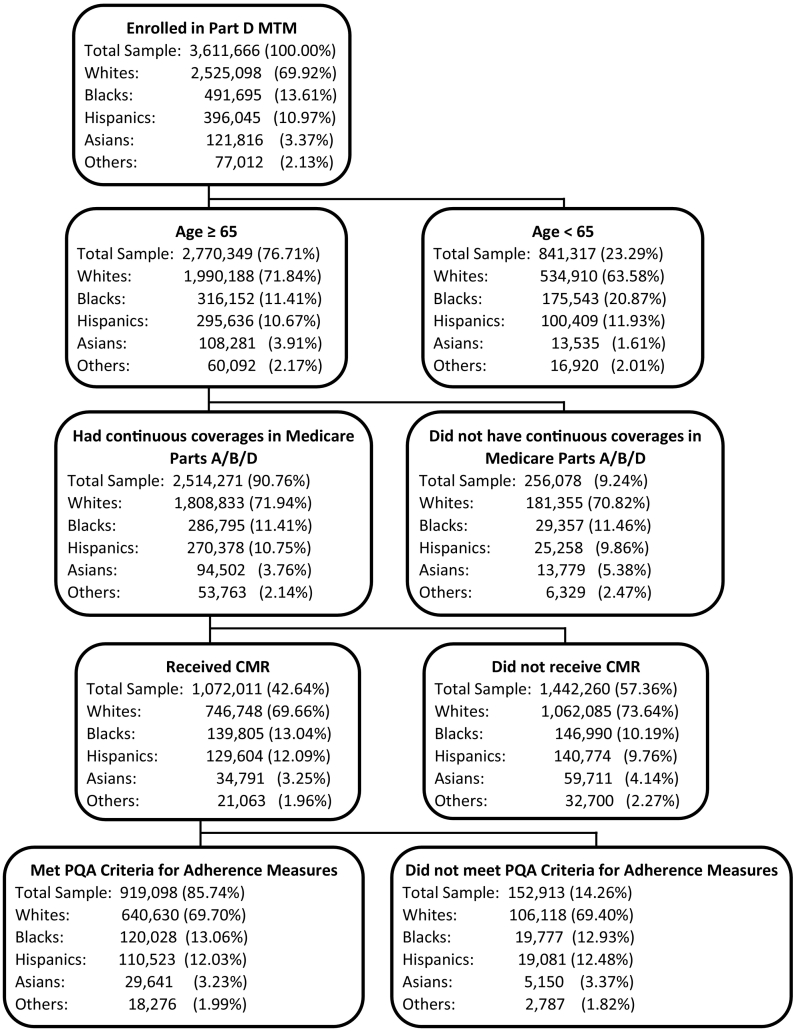
Numbers of beneficiaries in total sample and by race/ethnicity after each inclusion/exclusion criterion.

The analytic sample consisted of 919,098 Medicare CMR recipients who met the inclusion criteria. Among them, more than two-thirds were Whites (69.70%), followed by Blacks (13.06%), Hispanics (12.03%), Asians (3.23%), and Other (1.99%). Fig. 1 presents the numbers of beneficiaries dropped after each inclusion/exclusion criterion was applied. After the age criterion, the proportions of Whites, Asians, and Other respectively increased while the proportions of Blacks and Hispanics decreased. The racial/ethnic distribution remained about the same after the continuous coverage criterion. In contrast, applying the CMR receipt criterion led to a decrease in the proportions of Whites, Asians, and Other and an increase among Blacks and Hispanics. Such a distribution pattern remained about the same when the final sample was constructed.

Table 1 presents beneficiary characteristics by race/ethnicity. Compared to racial/ethnic minorities, Whites were older (except Asian), had a higher proportion of males (except Asians and Other), lived in counties with a higher proportion of married-couple families, a higher proportion of people having at least high school education, having lower income per capita as well as a lower proportion of people without health insurance (except Asians and Other). Whites were also less likely to live in an MSA or HPSA. Additionally, they were likely to have worse health status. Racial and ethnic groups had different geographical distributions: more Whites, Blacks, and Hispanics lived in the South, while more Asians resided in the West. All characteristics were significantly different by race/ethnicity (*P* < .001).

**Table 1:** Beneficiary characteristics by race/ethnicity among recipients of comprehensive medication review.

Characteristics	Non-Hispanic Whites		Blacks		Hispanics		Asians/Pacific Islanders		Other	
	(*N* = 640,630, 69.70%)		(*N* = 120,028, 13.06%)		(*N* = 110,523, 12.03%)		(*N* = 29,641, 3.23%)		(*N* = 18,276, 1.99%)	
	Number	%	Number	%	Number	%	Number	%	Number	%
Predisposing factors										
Age, mean (SD)	75.72 (6.70)		74.59 (6.49)		75.12 (6.60)		76.28 (6.96)		72.80 (5.91)	
Male	275,624	43.02	36,131	30.10	41,407	37.46	13,961	47.10	10,076	55.13
Proportion of married-couple families, mean (SD)^⁎^	0.74 (0.06)		0.66 (0.08)		0.67 (0.08)		0.70 (0.07)		0.72 (0.07)	
Proportion of education ≥ high school, mean (SD)^⁎^	0.88 (0.05)		0.86 (0.05)		0.82 (0.07)		0.85 (0.05)		0.87 (0.05)	
Income per capita (in $1000), mean (SD)^⁎^	48.78 (14.14)		50.22 (18.33)		52.90 (26.82)		64.02 (27.08)		52.91 (17.87)	
Proportion of no insurance, mean (SD)^⁎^	0.09 (0.04)		0.11 (0.05)		0.13 (0.07)		0.09 (0.04)		0.09 (0.04)	
Enabling factors										
Metropolitan statistical area^⁎^	516,600	80.64	107,277	89.38	106,242	96.13	29,193	98.49	15,933	87.18
Health professional shortage area^⁎^	585,897	91.46	115,307	96.07	108,661	98.32	28,786	97.12	17,308	94.70
Census regions^⁎^										
Northeast	138,513	21.62	23,665	19.72	28,575	25.85	8313	28.05	5219	28.56
Midwest	170,558	26.62	20,617	17.18	4238	3.83	1760	5.94	3652	19.98
South	222,163	34.68	67,057	55.87	47,476	42.96	4926	16.62	4644	25.41
West	109,396	17.08	8689	7.24	30,234	27.36	14,642	49.40	4761	26.05
Need factor										
Risk adjustment summary score, mean (SD)	1.28 (1.33)		1.15 (1.16)		0.98 (0.97)		1.11 (1.07)		1.15 (1.23)	

*Note:*^⁎^ indicates a county-level characteristic.

All characteristics were different by race/ethnicity (*P* < .001).

Abbreviation: SD = standard deviation.

**Fig. 2:**
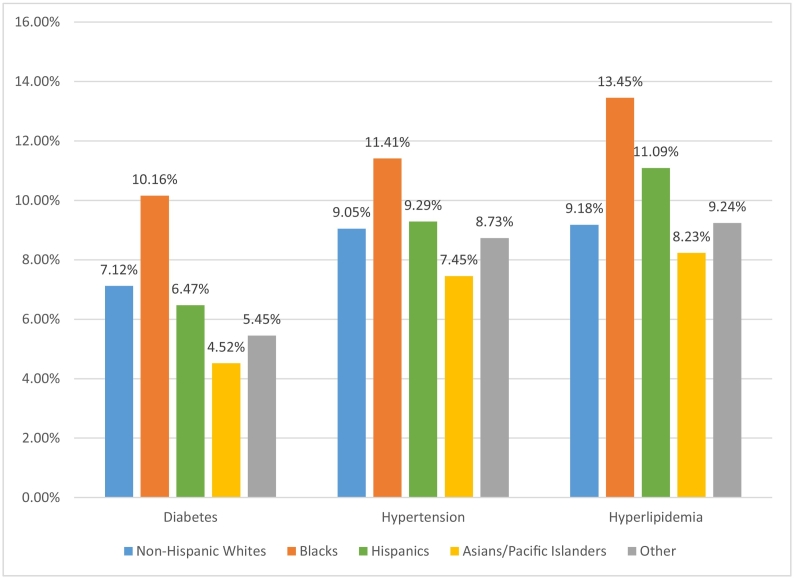
Proportions of medication nonadherent beneficiaries by condition and race/ethnicity among recipients of comprehensive medication review in 2017. Note: Difference across racial/ethnic groups for each condition of interest was significant based on a Chi-square test (*P* < .0001).

Unadjusted comparison of differences in outcome measures across racial/ethnic groups indicated that Blacks had higher proportions of nonadherence to medications for all 3 diseases of interest relative to Whites (Fig. 2). Specifically, the difference was 3.04% for diabetes (10.16% versus 7.12%), 2.36% for hypertension (11.41% versus 9.05%), and 4.27% for hyperlipidemia (13.45% versus 9.18%). Hispanics likewise had higher proportions of nonadherence to medications for hypertension and hyperlipidemia compared to their White counterparts, with the respective difference being 0.24% (9.29% versus 9.05%) and 1.91% (11.09% versus 9.18%). In contrast, Asians had lower proportions of nonadherence to medications for all 3 diseases of interest compared to Whites, as did Other for diabetes and hypertension. The latter had a higher proportion of nonadherence to hyperlipidemia medications compared to Whites (9.24% versus 9.18%). All differences across racial/ethnic groups for each disease of interest were statistically significant (*P* < .0001).

### 3.1 Multivariate regression results

Table 2 presents the adjusted logistic regression results of racial/ethnic disparity in nonadherence by medication. Compared with Whites, Blacks were respectively associated with a 39% (odds ratio or OR = 1.39, 95% confidence interval or CI = 1.33–1.45), 27% (OR = 1.27, 95% CI = 1.22–1.32), and 43% (OR = 1.43, 95% CI = 1.39–1.47) increase in the odds of nonadherence to medications for diabetes, hypertension, and hyperlipidemia. Likewise, Hispanics were associated with a 20% (OR = 1.20, 95% CI = 1.14–1.27) increase in the odds of nonadherence to hyperlipidemia medications. Conversely, the group was associated with a 10% (OR = 0.90, 95% CI = 0.85–0.96) decrease in the odds of nonadherence to diabetes medications compared with Whites. Asians were likewise associated with lower odds of medication nonadherence relative to Whites, except that the decrease was found across all 3 diseases. Specifically, the reduction in the odds was 37% (OR = 0.63, 95% CI = 0.57–0.70), 18% (OR = 0.82, 95% CI = 0.78–0.87)), and 7% (OR = 0.93, 95% CI = 0.89–0.98) for diabetes, hypertension, and hyperlipidemia medications, respectively. Additionally, Other were associated with a 21% (OR = 0.79, 95% CI =0.70–0.90) decrease in the odds of nonadherence to diabetes medications compared with Whites. All other adjusted between-group differences were not statistically significant.

**Table 2:** Adjusted logistic regression results of racial/ethnic disparity in nonadherence by medication among recipients of comprehensive medication review (standard errors clustered at county level).

Characteristics	Diabetes		Hypertension		Hyperlipidemia	
	(*N* = 310,779)		(*N* = 670,750)		(*N* = 765,990)	
	OR	95% CI	OR	95% CI	OR	95% CI
Predisposing factors						
Race/ethnicity						
Blacks	1.39	1.33–1.45	1.27	1.22–1.32	1.43	1.39–1.47
Hispanics	0.90	0.85–0.96	1.03	0.96–1.11	1.20	1.14–1.27
Asians/Pacific Islanders	0.63	0.57–0.70	0.82	0.78–0.87	0.93	0.89–0.98
Other	0.79	0.70–0.90	0.98	0.92–1.05	1.03	0.97–1.09
Age	1.01	1.00–1.01	1.00	1.00–1.00	0.99	0.99–1.00
Male	0.82	0.79–0.85	0.97	0.95–0.99	0.85	0.83–0.86
Proportion of married-couple families^⁎^	0.47	0.35–0.64	0.69	0.53–0.90	0.63	0.51–0.78
Proportion of education ≥ high school^⁎^	1.96	1.24–3.10	0.78	0.53–1.15	0.74	0.53–1.03
Income per capita (in $1000)^⁎^	1.00	1.00–1.00	1.00	1.00–1.00	1.00	1.00–1.00
Proportion of No Insurance^⁎^	1.34	0.79–2.26	0.98	0.60–1.60	1.00	0.68–1.46

Enabling factors						
Metropolitan statistical area ^⁎^	1.03	0.98–1.07	0.98	0.95–1.02	0.98	0.95–1.01
Health professional shortage area ^⁎^	0.98	0.92–1.05	1.02	0.98–1.06	1.02	0.99–1.06
Census regions^⁎^						
Midwest	1.09	1.02–1.17	0.97	0.93–1.01	1.01	0.96–1.05
South	1.25	1.16–1.33	1.07	1.02–1.11	1.10	1.05–1.16
West	1.23	1.14–1.32	1.04	0.99–1.09	0.97	0.92–1.01

Need factor						
Risk adjustment summary score	1.16	1.14–1.17	1.20	1.19–1.21	1.12	1.11–1.12

*Note:*^⁎^ County-level characteristic.

Reference groups: Whites, female, non-metropolitan statistical area, non-health professional shortage area, and Northeast region

Abbreviations: OR = odds ratio; CI = confidence interval.

Apart from the main findings on racial/ethnic disparity, several covariates were found to be consistently significantly associated with nonadherence (Table 2). For instance, being male and living in counties with higher proportion of married-couple families were both associated with lower odds of nonadherence, whereas living in the South and having a higher risk adjustment summary score, which suggested worse health status, were positively associated with nonadherence.

### 3.2 Decomposition results

As illustrated in the Methods section, this study only considered as disparity when racial/ethnic minority groups experienced higher odds of nonadherence than Whites; therefore, decomposition was performed for the 4 pairs of statistically significant disparities identified from the multivariate regression results, namely Black-White (B–W) disparity in nonadherence to medications for diabetes, hypertension, hyperlipidemia, and Hispanic-White (H–W) disparity for hyperlipidemia. Results are reported in Table 3.

**Table 3:** Decomposition of Black-White and Hispanic-White disparities in medication nonadherence among recipients of comprehensive medication review (in percentage points unless indicated otherwise).

Measure	Black-White						Hispanic-White	
	Diabetes		Hypertension		Hyperlipidemia		Hyperlipidemia	
Average nonadherence rate for Whites (%)	7.1233		9.0547		9.1834		9.1834	
Average nonadherence rate for titled minority group (%)	10.1553		11.4124		13.4467		11.0861	
Total absolute disparity	3.0320		2.3577		4.2633		1.9027	

Covariate contribution	Absolute	%	Absolute	%	Absolute	%	Absolute	%
Predisposing factors								
Age	−0.0307⁎	−1.01	−0.0047	−0.20	0.0499⁎	1.17	0.0270⁎	1.42
Male	0.2080⁎	6.86	0.0346⁎	1.47	0.1898⁎	4.45	0.0861⁎	4.53
Proportion of married-couple families^a^	0.3825⁎	12.61	0.2298⁎	9.75	0.2977⁎	6.98	0.2642⁎	13.89
Proportion of education ≥ high school^a^	−0.0920⁎	−3.03	0.0419	1.78	0.0553	1.30	0.1546	8.13
Income per capita (in $1000)^a^	−0.0090	−0.30	−0.0040	−0.17	−0.0057	−0.13	−0.0171	−0.90
Proportion of no insurance^a^	0.0277	0.91	−0.0025	−0.11	−0.0002	−0.01	−0.0006	−0.03
Enabling factors								
Metropolitan statistical area^a^	0.0145	0.48	−0.0119	−0.50	−0.0143	−0.34	−0.0262	−1.38
Health professional shortage area^a^	−0.0075	−0.25	0.0076	0.32	0.0104	0.24	0.0146	0.77
Census regions^a^	0.1134⁎	3.74	0.1164⁎	4.94	0.2064⁎	4.84	0.0316⁎	1.66
Need factor								
Risk adjustment summary score	−0.1999⁎	−6.59	−0.2267⁎	−9.62	−0.1553⁎	−3.64	−0.3309⁎	−17.39
Total explained by covariates	0.4070	13.42	0.1805	7.66	0.6339	14.87	0.2034	10.69

⁎ Significant at *P* < .05.

a County-level characteristics.

The difference between the average nonadherence rates for Whites and the minority group represented total disparity, which was 3.0320, 2.3577, 4.2633, and 1.9027 percentage points (pp.) for B–W diabetes, B–W hypertension, B–W hyperlipidemia, and H–W hyperlipidemia, respectively. With regards to individual covariate's contribution to the disparity, the biggest contributor across all 4 pairs was the proportion of married-couple families, which explained 0.2298 to 0.3825 pp. (or 6.98% to 13.89%) of the total disparity. For example, the disparity estimate of this covariate for B–W diabetes indicated that, if Whites had a similar distribution of the proportion of married-couple families in their county of residence as Blacks, the average nonadherence rate for Whites would increase by 0.3825 pp. or 12.61%. In other words, if Whites and Blacks were equalized on that characteristic, the disparity would be reduced by 0.3825 pp. or 12.61%. The next top 2 contributors across all 4 pairs of disparity included male gender and census region, with the former explaining 1.47% to 6.86% and the latter 1.66% to 4.94% of the difference. Additionally, age accounted for a small portion of the gaps for B–W hyperlipidemia (1.17%) and H–W hyperlipidemia (1.42%).

Among the covariates, risk adjustment summary score made the biggest negative contribution across all pairs of disparity. Specifically, it accounted for −6.59% for B–W diabetes, −9.62% for B–W hypertension, −3.64% for B–W hyperlipidemia, and −17.39% for H–W hyperlipidemia. For instance, the estimate for the last pair of disparity suggested if Whites had a similar distribution of risk adjustment summary score as Hispanics, the average nonadherence rate for Whites would decrease by 17.39%, which meant that the gap would be widened by 17.39% if the 2 groups were equalized on that characteristic. The other negative contributors were only found in B–W diabetes, including age (−1.01%) and proportion of people having at least high school education (−3.03%). All other covariate contributions were not statistically significant. Overall, the total portion of disparity explained by all covariates was 13.42%, 7.66%, 14.87%, and 10.69% for B–W diabetes, B–W hypertension, B–W hyperlipidemia, and H–W hyperlipidemia, respectively.

## 4 Discussion

Findings from the multivariate regression analysis indicated that, compared with their White counterparts, Black Medicare CMR recipients were more likely to be nonadherent to medications for diabetes, hypertension, and hyperlipidemia while Hispanics were more likely to be nonadherent to hyperlipidemia medications. Thus, racial disparities between Whites and Blacks persisted to a larger degree than ethnic disparities between Whites and Hispanics after confounders were controlled for. The differential disparity pattern was consistent with the pattern documented in access literature which noted that, relative to other minorities, Blacks experienced more severe disparities in health care access,^33^ one of the major determinants of medication adherence.^4^

Decomposition analysis further revealed that the observed characteristics included in the regression model explained the maximum close to 15% of the identified disparity, with B–W hypertension explained the least (7.66%) and B–W hyperlipidemia the most (14.87%). Across all 4 pairs of disparity, covariates that accounted for most of the gaps included the proportion of married-couple families in beneficiaries county of residence, census region, and male gender. Some other covariates such as age also yielded statistically significant estimates; however, because the absolute difference that they made was less than 0.10 pp., those estimates may not have real-world significance.

The biggest contributor, proportion of married-couple families in the county of residence, may be considered as a proxy for community affluence and social support. It is worth noting that this covariate appears to have overpowered other measures for community socioeconomic composition such as per capita income and proportion of uninsured population. The association between population affluence/socioeconomic status and nonadherence has been widely documented.^1^^,^^34^ An integral component in the conceptual model of medication adherence determinants,^4^ social support has been found to be positively associated with adherence among hypertensive Blacks.^35^^,^^36^ It may also help alleviate depression, which is pervasive among people with chronic conditions and has been reported to negatively impact adherence.^37^^,^^38^ The descriptive statistics of this study sample showed that both Blacks (66%) and Hispanics (67%) lived in counties with a lower proportion of married-couple families than Whites (74%), suggesting that a lower level of social support may contribute to the higher nonadherence rates for the 2 minority groups.

Census regions, another major contributor to the disparity, may capture geographic differences in barriers to medication adherence. Geographic location has been observed to be associated with diabetes, hypertension, and hyperlipidemia medication adherence, with the South generally found to have a lower adherence rate.^39^ For instance, South Atlantic, East South Central, and West South Central were the bottom 3 for PDC measuring statin adherence, relative to other regions in the country.^39^ In the present study's analytic sample, the regional distribution patterns of Blacks and Hispanics were markedly different from that of Whites: Over half of Blacks (55.87%) and nearly half of Hispanics (42.96%) lived in the South while only about one-third of Whites (34.68%) did so. Southern states are more likely to be burdened by adverse health outcomes^40^ and some of them tend to be less wealthy; the governments may have more budget constraints that impact health care resources available to their residents. Nonadherence due to higher disease burden,^41^^,^^42^ lack of access to pharmacy or prescription medication coverage has been well documented in literature examining barriers to adherence.^1^^,^^34^

In addition to census regions, male gender made a sizeable contribution to the disparity. Blacks (30.10%) and Hispanics (37.46%) had significantly lower proportions of males than Whites (43.02%). Whereas evidence on gender as a predictor of nonadherence is mixed,^34^ this study's multivariate regression results suggested that male gender was associated with a lower odds of medication nonadherence relative to female gender. Previous studies of similar findings reasoned that it may be because women tend to take care of others more than of themselves.^43^^,^^44^

The decomposition results indicated that at least 85% of the disparity was not explained by the covariates included in our regression model. The large unexplained portion attested to the fact that medication adherence is a complex issue influenced by multiple factors, such as those related to patients, medications, providers, and health systems,^1^^,^^4^ and not all of them are easily measurable. Some of the factors that likely explained the disparity but were not captured by our regression model include predisposing factors such as health belief, cultural background, level of trust in the health care system,^4^^,^^33^^,^^45^ and enabling factors such as the extent of provider attitude and service influenced by stereotype and prejudice.^46^ The complexity of medication adherence requires a multi-faceted approach to improve adherence,^4^ a rationale embraced by the Medicare Part D MTM program that was designed to bridge across stakeholders at different levels including beneficiaries, their caregivers, healthcare providers such as pharmacists and physicians, Part D plans, and CMS.

As a required component of the MTM program, CMR is provided annually and its completion rate is a Star Ratings quality measure, calculated as the percentage of MTM-enrolled beneficiaries who receive a CMR in the reporting year.^8^ Whereas the measure certainly motivates plans to increase their rate of CMR completion, it is unknown the quality of the CMR service received by the beneficiary or whether any medication management plan developed during the CMR is indeed followed through. Provider-patient communication has been identified as a key factor in adherence,^47^ and provider communication perceived by patients as understanding and collaborative has been positively associated with medication adherence.^48^^,^^49^ It is therefore crucial for a provider to consider a patient's social profile, such as cultural background and health belief, when delivering a CMR. Metrics on beneficiary experience with CMR delivery may be included in Star Ratings so that not only the quantity but also the quality of this component is evaluated, allowing it to play a more meaningful role in addressing the complex issue of medication adherence. Data on beneficiary experience with CMR delivery may be captured in the form of a survey linkable to the current data that solely reflect the date of CMR receipt.

The present study had several limitations. First, the use of Medicare claims data limited the types of individual-level socioeconomic covariates available for our analyses. Although the claims data were supplemented with community-level variables obtained from AHRF, those variables might not be the perfect proxies. For instance, income and insurance were found to be the top 2 contributors to racial/ethnic disparities in healthcare access,^50^ which affects medication adherence.^1^^,^^34^ However, this study did not find a statistically significant relationship between these 2 county-level variables and the outcome measures in the multivariate regression analysis, neither were they found to account for the identified disparities in the decomposition analysis. The fact that the covariates in the existing regression model explained only a small percentage of the total disparity might further testify to the limitation of studies based on claims data analysis. Second, the outcome measures were constructed by calculating a beneficiary's PDC based on fill records. There was no indication whether the beneficiary in fact ingested the medications or did so according to prescription records. However, the PDC-based adherence measure is endorsed by PQA and has undergone rigorous testing. The measure has been not only adopted by CMS as a core metric in its Star Ratings system but also widely used in adherence research. Finally, the Gelberg-Andersen Behavioral Model used in the study might have its limitation as a theoretical framework for racial/ethnic disparities decomposition, given that the covariates only explained a small portion of the total disparity. While the use of community-level covariates might contribute to the large unexplained portion, more than 1 decomposition study using individual-level survey data and the same Andersen's model to examine racial/ethnic disparities in medication use found that the model did not explain most of the disparities.^51^^,^^52^ This testifies to the complexity of health disparities and solutions to address them.

To the authors' best knowledge, the study was the first that used 100% of the most recent Medicare claims data to examine racial/ethnic disparities in medication adherence among CMR recipients. Applying inclusion criteria to the entire Medicare population afforded the study a large sample size and great statistical power. In addition, the decomposition analysis provided initial evidence of the potential sources of the disparity and the following policy implications. First, the contribution of married-couple families to the disparity confirms the important role of social support. Hence, more community-based support programs may be needed to effectively reduce the racial/ethnic disparities in adherence. Second, the significant contribution of census regions and male gender suggests that interventions tailored to geographic and gender differences may be more effective than a one-size-fits-all approach. Due to the limitation of claims data, the identified sources of disparity are far from exhaustive. More research is warranted to explore such sources by triangulating findings based on claim data with those derived from survey data and using qualitative methods. When data on beneficiary experience with CMR delivery become available, research may shed more light onto the quality of CMR such as how providers identify and engage vulnerable CMR recipients.

Medication adherence is a complex behavior. Many potential adherence determinants that are beyond the scope of this study deserve attention in future racial/ethnic disparity research. Some examples of these determinants include racial/ethnic differences in the date of first prescription fill, having a 90-day versus a 30-day supply for the medications, drug classes, brand versus generic medications, or out-of-pocket costs for the medications. Given that diabetes, hypertension, and hyperlipidemia are often comorbidities of each other, future studies may investigate how comorbidity should be factored in medication adherence measures so that patients taking multiple medications for one condition and medications for multiple conditions can be appropriately accounted for. Another measure-related direction for future studies may be conceptualizing the PDC-based adherence measure as a continuous, instead of dichotomous, variable to examine the extent of adherence.

Besides examining adherence determinants and alternative adherence measures, future research may also explore different study designs to corroborate the findings from this study. For instance, the present study only investigated one year and considered CMRs received in that year. The PDC only considered medication fills that occurred after the CMR receipt. Thus, beneficiaries who received CMRs later in the year had a shorter treatment period for their PDC. Future studies may examine multiple years to determine whether and how the results differ from the findings of this study. Additionally, while this study followed the usual practice by using census regions as a covariate, future studies may use states instead that might better capture variations at the state level. Finally, this study used risk adjustment summary score as a proxy for health status. Researchers who have access to the MBSF Chronic Conditions Segment may use the first date of condition to approximate the duration of disease. Alternatively, the number of CMRs received relative to the number of MTM-eligible years may be used as a proxy for perceived need.

In conclusion, among older Medicare Part D MTM enrollees who received a CMR in 2017, relative to Whites, Blacks were more likely to be nonadherent to diabetes, hypertension, and hyperlipidemia medications while Hispanics were more likely to be nonadherent to hyperlipidemia medications. The observed characteristics included in the regression model accounted for the maximum close to 15% of the identified disparity, most of which was explained by the proportion of married-couple families in beneficiaries county of residence, census region, and male gender. These findings suggest that a lower level of community affluence and social support, regional variations, and a lower proportion of males in Blacks and Hispanics may contribute to their higher nonadherence rates. The large unexplained portion of the disparity attested to the fact that medication adherence is a complex issue that requires a multi-faceted intervention. Measures of beneficiary experience with CMR delivery may be included in Star Ratings for Part D plans in order for CMR to make a more meaningful impact in addressing the complex medication adherence issue.

## Funding

Research reported in this publication was supported by the 10.13039/100000002National Institute on Aging of the National Institutes of Health under award number R01AG040146. The content is solely the responsibility of the authors and does not necessarily represent the official views of the National Institutes of Health.

## Declaration of Competing Interest

Xiaobei Dong: None. Chi Chun Steve Tsang: None. Jim Y. Wan: None. Yachen Tina Shih: None. Marie A. Chisholm-Burns: Received funding from Carlos and Marguerite Mason Trust. Samuel Dagogo-Jack: Led clinical trials for AstraZeneca, Boehringer Ingelheim, and Novo Nordisk, Inc., received consulting fees from AstraZeneca, Boehringer Ingelheim, Janssen, Merck & Co. Inc., and Sanofi, and has equity interests in Jana Care, Inc. and Aerami Therapeutics. William C. Cushman: Received grant funding from Eli Lilly. Lisa E. Hines: None. Junling Wang: Received funding from 10.13039/100006483AbbVie, Curo, 10.13039/100008021Bristol Myers Squibb, United States, 10.13039/100004319Pfizer, and Pharmaceutical Research and Manufacturers of America (PhRMA), and serves on Heath Outcomes Research Advisor Committee of the PhRMA Foundation.
